# Peculiar Case of Diaphragmatic Hernia

**Published:** 1853-07

**Authors:** Henry I. Bowditch

**Affiliations:** Member of the Boston Society for Medical Observation


### BUFFALO MEDICAL JOURNAL

AND

### MONTHLY REVIEW.

VOL. 9. JULY, 1853. NO. 2.

### ORIGINAL COMMUNICATIONS.

ART. I.—*Peculiar case of Diaphragmatic Hernia, in which nearly the whole of the left side of the diaphragm was wanting, so that the stomach and a great part of the intestines lay in the left pleural cavity; compressing the left lung, and forcing the heart to the right side of the sternum. This condition, evidently congenital, existed in a man who died at the Massachusetts General Hospital, with Fracture of the Spine, caused by a heavy blow upon it. To which is added an analysis of most, if not all of the cases of diaphragmatic hernia found recorded in the annals of Medical Science.* By HENRY I. BOWDITCH, Member of the Boston Society for Medical Observation. Presented to the Society in 1847.

(Concluded.)

### II. SYMPTOMS DURING THE FATAL ATTACK.

#### CEPHALIC SYMPTOMS.

They are noticed five times.

*Convulsions* were observed in a child who died a few hours after birth. The extraordinary dyspnœa seemed to be the cause of it. At the autopsy, a considerable portion of the liver was found in a hernial sac. This symptom was seen likewise in the case of a man, in whom death occurred in a few hours, after having been preceded by the "greatest degree of dyspnœa." In him the hernia consisted of the colon, omentum, and pancreas, with blood from rupture of the pancreatic vein.

*Grinding of the teeth* was noticed in the case of a child, who died in twelve hours, and in whom the stomach and omentum only were in the chest.

*Delirium* occurred in but a single case; and in that, I think it may have been more owing to a comminuted fracture of the leg than to the hernia.

There was *numbness*, without paralysis of the legs, in one case.

I think we may safely infer that cerebral symptoms are uncommon in this affection. The result above obtained corresponds—1st. With the fact that authors have never examined the head after death, which they certainly would have done sometimes, if any serious symptoms had occurred; and 2d. With what I have stated above, when treating of symptoms that occur previous to the fatal attack.

### THORACIC SYMPTOMS.

*(During the fatal attack.)*

Noticed in thirty-six cases.

### PULMONARY SYMPTOMS.

Of the thirty-six cases in which mention is made of any pulmonary symptoms, in twenty-seven there was some *labor in the respiration.* This large proportion proves that some modification of labored breathing must take place in at least three-fourths of all affected with diaphragmatic hernia, and I am inclined to believe that this is a small proportion, because from our previous investigations we have seen—1st. That the lungs are compressed in a larger proportion of times, and I cannot conceive of there being so much pressure without a corresponding dyspnœa. 2d. The results, upon this point, in our investigation of the antecedent symptoms confirm this view of the case. We meet, in our daily practice, with cases of dyspnœa which, at the first glance, are not very obvious; it is rather breathlessness than dyspnœa, and I presume some such cases have been omitted.

This symptom varied in its degree. It is described in one case as orthopnœa; as the greatest dyspnœa, or dyspnœa; breath interrupted; frequent and labored; very imperfect; suffocative on lying down; much oppressed, confined; panting; short and quick; in the other cases.

I endeavored to find out if possible, as when looking at this question previously, whether this symptom in its different degrees of severity bore any relation to the amount of disease in the diaphragm, or of the hernia of the abdominal organs into the chest, but I found it to be impossible. It seems to depend on causes which are not indicated in the cases. More thoroughly detailed cases may elicit more on this subject than can be done at present.

In one case, the respiration is said to have been always *free*. It was the case of a drunkard, and he seems to have sunk with vomiting and purging after immoderate drinking, and possibly may not have died so much of the hernia as of his bad habits.

### COUGH.

This symptom is spoken of in only eight of the thirty-six cases. Possibly in some cases the record of it is omitted, although this symptom really occurred. In fact, I can scarcely believe that it should occur so seldom, although this result is similar to that which we obtained from examination of antecedent symptoms. We have examples, however, of a similar absence of cough in various diseases, involving the pleura and parts adjacent to the lungs. Pleurisy, for example, very frequently produces very little cough. It seems to me that we should be allowed to deduce, (1st. From the infrequency of its being noticed in diaphragmatic hernia; and 2d. From the character of the symptom when recorded,) that as a symptom, diagnostic of the disease, it is of very little importance. The cough was said to be frequent in three cases; and in one case, there was an inability to cough, owing to pain being caused by it. In other cases it was noted without any qualification.

### EXPECTORATION.

This symptom confirms what we have said in regard to the cough. It was mentioned but three times and always as having occurred after a fall. In two, in which extravasations of blood were found in the abdomen, a little blood was raised, but as the lungs were found simply compressed I doubt whether it came from the pulmonary structure. In the third there was a copious raising of mucus. May we not infer that, if this symptom does occur more frequently, it is nevertheless rather an accidental than necessary accompaniment?

### HICCOUGH.

This was noticed eleven times, or in nearly one third of the cases, and becomes, therefore, an important symptom of the affection. It was usually frequent and severe. In one case it existed during the last nine days of the patient's lif·, and was constant; in another it was loud and the immediate precursor of death. It sometimes resembled sobbing, and in one case, a child was born, sighed three-fourths of an hour, and expired.

In examining it with reference to the morbid anatomy, I found it occurred twice in cases in which the right side was ruptured, nine times in hernia of the left. I did not find this sign connected with rupture of any particular structure of the diaphragm, or hernia of any particular number of organs, or bulk; for, in one case, there was only a small part of the ileum in the aperture, and in another were the greater part of the small intestines, omentum, spleen, and pancreas; while the remainder of the cases presented every variety between these two.

I might cite, as another reason for admitting this as an important sign in this affection, the well known fact that hiccough is generally considered one of the symptoms of any disease of the diaphragm.

### PAIN IN THE CHEST AND SIDES.

In regard to this symptom I refer the reader to the article "Pains, &c., in the abdomen.

### VOICE.

In one case, (of a child that died half an hour after birth,) I find recorded that there was difficulty of breathing from birth, that some time elapsed before the patient cried, and that when it did so, there was a peculiar note to the voice. I know not whether the very peculiar state of the parts, found after death, will explain this symptom, but the coincidence is a curious one. In this case alone have I found that the colon, full of meconium, was pushed up between the aorta and spine, and was pressed upon the trachea as high as the first rib.

### PHYSICAL SIGNS.

These signs have been almost wholly neglected. Laennec* has stated that they would prove of much service in this affection, but, as he had never seen a case, he merely makes the suggestion that it may be recognized by absence of the respiratory murmur and the presence of borborygmi in the chest. Mr. Lawrence, so far as I can discover, is the only person who ever, (before our

* Traité de l'Auscultation Médiate. Paris.

case at the hospital,) recognized the hernia by these signs,* or, as I may add, by any other save by the morbid appearances after death. It may have been suspected, but never definitely diagnosed.

I proceed now to name them, and under each head I shall, in addition to giving the usual analysis of facts, which in this particular will be very meagre, make suggestions in regard to some that *must* have been present though not noticed.

*Inspection.* This was used five times, including our own case. In three the chest was dilated; a roundness, prominence of the affected side being marked in some. In one case the cartilages seemed luxated inward; and in another, the abdominal parietes were drawn up and down with each inspiration. In our case, there was a quietness of the whole side. I may allude here to the peculiar symptom described above, viz., a "crawling round the ribs," which, although I have not much confidence in its being observed frequently, may, however, occasionally occur.

So much for the records of science. But may we not step now beyond these narrow limits and declare that, according to known physical laws, there *must be* in the majority of cases, a prominence and a partial or complete immobility of the side of the chest in which the hernia exists? In looking at Table 10, I find that in fifty-five out of the eighty-eight cases the lungs were *much* compressed, by which term it is meant that the stomach or intestines, or other of the abdominal viscera, occupied the place of a major part of the lung. If we remember that (by Table 9) only in four cases of seventy-nine was there a single small organ in the chest, but that in seventy-three cases out of the same seventy-nine, either the stomach or some of the intestines were there, with some one or more organs; if we recollect likewise that the portions of the alimentary canal, when thus incarcerated, often become much distended, we can readily believe that the side of the thorax will be distended and partially immovable. This prominence and immobility must likewise be most manifest in front, because the lung would tend to be pushed up and backward in consequence of its natural attachments to the spinal column. These considerations induce me to believe that in a great majority of the future cases of this kind, prominence and immobility of the thorax will be important aids to diagnosis.

*Palpation.* I am not aware that this was used at all except, perhaps, to recognize the changed position of the heart. Yet as physical laws exist there must be a different vocal fremitus on the two sides of the chest, when

---

* London Lancet, Sept. 5, 1835.

the lungs are forced back by an intestine distended with gas, and the walls of the chest are thereby more expanded than usual. What difference there should be I cannot say. That a difference would exist I have no doubt.

I have alluded to the use of this method in recognizing the changed position of the heart, but as auscultation will do this more effectually, I must refer to that.

*Mensuration* might be used very frequently, and as usefully as we employ it in pneumo-thorax, pleurisy, &c., but I do not think it has ever been used, and probably in cases of recent rupture, we shall generally be unwilling to use it, owing to the sufferings of the patient.

*Auscultation.* This was used in six, possibly in seven cases. I likewise recognized the affection chiefly by the use of this mode of examination.

Mr. Lawrence* found little or no *respiratory murmur* on the affected side. In this case a large part of intestines, the whole omentum, part of the spleen and the whole pancreas compressed the lungs to the size of a man's fist.

Dr. Reid † found "a respiratory murmur" in the left side in his case, and yet there were more than six pounds of fluid with one foot of colon and omentum in the pleural cavity. It is to be regretted that Dr. R. did not give a more detailed account of this case, and one is led to doubt whether the sound may not have been transmitted from the other lung; because the lung of the diseased side was found pressed back against the spine and attached to the diaphragm and "containing no air." Possibly the sound may have resembled that heard in cases of large effusions into the chest.

In our case, and one other, there was *absence of the respiratory murmur* on the diseased, with puerile respiration on the healthy side. But in our case, when the patient took a deep inspiration, the murmur was heard perfectly vesicular and pure to the second rib. A full breath increased certain *borborygmi*, which had taken the place of the respiratory murmur below this point. To the inexperienced ear they seemed to form so completely a part of the act that they were, at times, supposed to be in the lungs. But they had exactly the characteristics of the sounds heard over the abdomen in cases of intestinal disturbance, a mixture of gurgling, whistling and blowing, and though excited at times by the act of respiration, they could be heard, even when the patient held his breath. It seemed as if the distention of the lung excited the vermicular motion. But this was not always the case, because often I could not excite them, either by a forced cough or inspiration.

---

* Lancet, Sept. 5, 1835.

† Edinburgh Medical and Surgical Journal, Jan., 1840.

Occasionally a *metallic tinkling* was heard, such as we sometimes hear over the stomach. One day, an amphoric sound was heard, quite intense, over the cartilages of fourth and fifth ribs, seeming to be stomachic though affected slightly by the act of inspiration. Might we not excite these various sounds (borborygmi, &c.) by making the patient drink some stimulating liquid??

The *voice* appeared natural in our case, and in one that occurred in Guy's Hospital. I do not find that it has been noticed in others. It deserves attention, for I believe some modification of it may often be present.

In three cases, a subcrepitant *râle* was heard, twice in the back, once in front and upper part of the back. Bronchophony, likewise, was heard in one. In all, pneumonitis was found at the autopsy, and these modifications of the respiratory murmur and voice were owing to this accidental circumstance.

It seems to me very probable that some auscultatory phenomena might be produced by pressing suddenly on the abdomen, and thus forcing air into the intestines while in the pleural sac; or by disturbing the contents of the intestines and thus causing peristaltic action.

The pulsations of the *heart* were noticed in four cases, and in three of them they were to the right of the sternum. In our hospital case, it was heard there likewise. These numbers give, I know, a very inadequate idea of the real number of times that the sounds will be heard out of their usual situation.

When treating of the pathological anatomy of the heart, I remarked on the probable frequency of this displacement. I would likewise refer now to what I stated when speaking of inspection; for the argument, drawn from Table 9, applies to this subject as well as to prominence of the chest. This dislocation of the heart must be considered an important element in the diagnosis.

May not I suggest that not infrequently a bellows murmur will be heard, owing to the constrained position of the heart? It has never been noticed, but were I to perceive it I should be very careful of inferring from its existence any serious lesion of the organ.*

*Percussion.* This method was used five times. There was dullness four

---

* Since writing this, I have seen a record of a case in which a souffle was observed about the roots of the bronchi. The heart in this case was pushed strongly to the left by a sac at the right side. (Bul. de la Societé Anatomique, Auzelly's Thesis.†)

† Thèse pour le doctorat en Médecine. Paris, 1842, par Aristide—Raymond Auzelly.

times at the back of the chest, in three of which there was either pneumonia or pleuritic effusion; and in the other, the liver, colon, and omentum, were in a sac. In one there was good sound, in which, though there was a slight degree of pneumonia, there were the stomach and colon to distend chiefly the left side of the thorax. Finally, in our case there was decided tympanites. This result, I believe, conveys a very inaccurate idea of the real use of percussion in this complaint. For in seven only of all the cases was there sufficient fluid to cause dullness; in only six were the lungs carnified or hepatized, &c. On the contrary, by reference to Table 9, we find that in seventy-two out of the eighty cases, either the stomach or some portion of the alimentary canal was in the chest. Now, although in some of these cases only a small portion of the canal was in the chest, or perhaps if in the thorax it was contracted,—this is not the usual result; but the stomach or intestines are distended with air. This distention must cause tympanites as in our case, and generally in front of the chest, especially at the left side of the same. There may be, also, a removal, toward the right, of the usual dullness observed over the heart, owing to the organ being pushed out of place.

### PULSE AND FEVER.

The pulse was noticed eleven times.

Its increased frequency was remarked in all but two of the cases. It was small or feeble in eight; tremulous in two; firm or hard in two; soft in one; strong and full in one; and intermitting in one. It has evidently been omitted in a majority of the records, and we cannot make any deductions, except, perhaps, that an increased frequency seems not uncommon, but of this we cannot be certain, because, as the time of the examination is not mentioned, except in two cases the record may have been made of the latest hours of life only.

*Fever* was spoken of but twice, but cold, clammy sweats were observed five times. This part of the subject needs further research.

The skin was cold and livid about the chest, abdomen or extremities, in six cases. It was covered with cold, clammy sweats, in three.

### ABDOMINAL SYMPTOMS, &C.

*(During the fatal attack.)*

Noticed in thirty-five cases.

*Tongue and throat, &c.* They were not mentioned but twice. In the case of the former it was clean and moist at one time, and only had a little brownish coat on another occasion.

In one case there was great *distress in the throat,* but the post-mortem appearances did not account for it.

Great *thirst* is mentioned in one case. A *grinding of the teeth* occurred in another. Anorexia and *cardialgia* occurred in a beer drinker, who died in consequence of an immoderate draught of his favorite liquor.

There was *rejection of food* before it reached the stomach, in one case. In this the stomach was wholly inverted in the pleura, so that food must have found access to it with difficulty.

### STOMACH.

*Nausea or retching*, or ineffectual efforts at vomiting, were noticed six times. I found that these symptoms were not particularly caused by the abnormal position of the stomach; at least, to produce them it was not necessary for the stomach to be confined in the chest, for, in two out of the three cases in which nausea was mentioned, the stomach was not above the diaphragm. In two cases of *ineffectual efforts at vomiting*,* the same general result was obtained. In one of Kirchbaum's cases, I find the expression —*weakness of the stomach*—which, I presume, indicates a similar symptom. In this case there was likewise vomiting, and the stomach had passed into the chest.

But the principal symptom in the disease, connected with the stomach, was *vomiting.* Mention is made of this twenty-two times. Add to these four cases of retching, &c., and four more of whom it was said that the individuals died of iliac passion, and who probably had vomiting, we have thirty cases out of thirty-five who had either a disposition to vomit or actual vomiting.

This, then, becomes a very important symptom for the diagnosis. In fact no other, save dyspnœa, can be compared with it. Their combination in any case, of course, is still more important. We shall discuss this point at a future time. (Vide Diagnosis.)

This symptom not merely existed; but in the majority (fourteen out of twenty-two) of the cases, it is recorded as severe, very severe, constant or as "vomiting of every thing."

The matters vomited were mentioned in eight cases. They were dark and

* Increasing very much the pains in the abdomen.

fœtid in three cases; it was a kind of purulent pap, acid and fœtid in one; a brownish mucus, probably altered blood, with sarcinula ventriculi in one; it was distinctly blood in one; and stercoraceous in two, toward end of life.

In one of the above which had altered blood, the matters vomited gave an acid reaction, had no taste, and was covered with a yeasty froth.

On comparing this symptom with the post-mortem appearances, I found that the stomach, in one case, was merely violently pressed up against the diaphragm; in three others, other abdominal organs were in the chest; in the remainder (eighteen in twenty-two) this organ was either wholly or in part in the chest.

### PAINS ABOUT THE ABDOMEN AND CHEST.

These pains were noticed seventeen times in the abdomen. In two cases they were described as great distress at precordia; in ten they were very severe, intolerable, excessive or violent pains or colics; in four they were chiefly limited to the left side; in two to the right, one of which of last had a dragging sensation at the right; in one it was confined to the upper part of the abdomen; in another, chiefly to hypochondrium. Finally, in one case, it was stated that there was no pain in the region of the stomach, but there was a violent pain at the lower part of the sternum.

Pains were "in the side" or chest in eight cases, but four of them were among the seventeen who had pain in the abdomen. These pains were generally severe and acute, and never recorded as dull.

In comparing these results with the post-mortem appearances, I have been foiled, as heretofore, but there seemed to be more severe and intolerable pains in those cases in which a small part of the intestines was firmly strangulated than in others, where there was a larger quantity of viscera in the chest. I cannot lay much stress on this point as the number of facts are too small and records too indefinite.

### ALVINE DISCHARGES.

Noticed eleven times.

*Costiveness*, usually of an obstinate character, was reported seven times. These prove that this symptom occurs frequently. But it is to be regretted that we do not find more detail in the cases given by authors, because I believe that the character and frequency of the alvine discharges would have presented interesting points for diagnosis and prognosis. Sir Astley Cooper says that in diaphragmatic hernia, we have the usual symptoms of internal strangulation, combined with those of asthma. As costiveness is very commonly the accompaniment of this strangulation, we must, I think, meet with this symptom oftener than our numbers would seem to indicate.

The bowels were loose, with fœtid dejections, in four cases; they were flatulent in one.

### ABDOMEN.

It was *tense and sore*, on pressure, in one case. This was one in which general but not severe peritonitis existed.

| | |
|---|---|
| It was *tympanitic* . . . . . . | in 2 cases. |
| " retracted . . . . . . | " 2 " |
| " tense . . . . . . . | " 1 " |
| " hard or rigid . . . . . | " 2 " |
| " depressed and drawn inward at each inspiration | " 1 " |

These facts are important from their very meagreness. It confirms the inferences which I drew when treating of the pathological condition of the abdomen. In the diagnosis of the disease it is of some importance for the practitioner to feel assured that, in the vast majority of the cases, there is no general peritonitis.

### URINE.

This was noticed only once. It was free, without blood; scanty, turbid from lithates, with purple sediment. The state of the kidneys are not mentioned in the autopsy.

### OMENTUM.

This was protruded through the abdominal parietes in one case, in which there were two wounds about the ensiform cartilage.

### SKIN AND GENERAL STATE OF PATIENT.

Mentioned in twenty-three cases.

Great lassitude or faintness in five cases. In two a wound had been inflicted. In one there was great depression after vomiting came on. In two cases, the patients were able to walk some distance after accidents which ruptured the diaphragm. One of them rode in a coach nearly 150 miles, without any complaint, though he became ill soon after his arrival, more than twenty-four hours after the accident.

That great lassitude should be likely to happen in case of so severe an accident as rupture of the diaphragm, seems very probable. I therefore doubt whether our numbers express very accurately the number of those who are prostrated by it. But it is extremely curious to know that a man is at times able to sustain himself for so long a period as twenty-four hours after a rupture, in which one-half the stomach was forced through the diaphragm and some blood effused.

### RESTLESSNESS, &C.

This feature of the affection was observed eight times. Once it was simple "discomfort." The left lung was in this case very much compressed. There was a starting and shuddering at birth, so great that "fits" were feared, and death took place in one and a half hours after birth. A third was unable to lie on the right side. In the remainder there was great restlessness, patients sometimes tossing about in agony at the intensity of their sufferings.

### COUNTENANCE.

The eyes were found open, haggard, or starting from their sockets, in three cases. All three cases were the results of severe falls. The face was pale in three cases; it had an anxious look in three more. It was expressive of great suffering in two; it was red, after having been livid previously, in one. After death it had a smiling aspect in one; it was natural in another; the veins of the forehead were distended, the commissures of the mouth were drawn apart showing the teeth, and the lips were covered with a bloody fluid in another; it was drawn to one side, as from paralysis, in one.

I think that we may infer from these few facts that the countenance frequently indicates the severity of the disease. The last two cases are the only ones in which I find notice of what some authors say is so very common in this and other affections of the diaphragm, viz., the sardonic smile. I do not believe that it is of such frequent occurrence as has been stated.

### INCIDENTAL APPEARANCES.

In ten other cases there were various conditions, not connected, save incidentally, with the hernia, viz., fractures, redness of skin, vesicular eruptions, &c., but of these I need not to speak.

### III. ORIGINAL CAUSES OF THE HERNIA.

I have made the following table for sixty-eight cases:

*Table* 11.

| | | |
|---|---|---|
| It was congenital | | in 26 cases. |
| It was from drunkenness and debaucheries | | " 3 " |
| " " | bullet wound | " 3 " |
| " " | stabs with sword or lance | " 9 " |
| " " | "wounds" | " 3 " |
| " " | absence of diaphragm | " 1 " |
| " " | falls | " 13 " |
| " " | labor pains | " 2 " |
| " " | sudden strain | " 1 " |
| " " | blows | " 4 " |
| " " | being run over | " 2 " |
| " " | fracture of the rib | " 1 " |
| | | 68 cases. |

By the above table we perceive that the congenital cases of diaphragmatic hernia are about one-third of the whole number. There is another class of cases of which, until the last year, we might have anticipated few examples in this country, viz., those in which the hernia is caused by stabs or bullet wounds; but, probably, among the military who have fallen on the plains of Mexico, we should find not a few pathological specimens of this kind. Some one or more of the survivors of that unholy war may be bearing even now the results of it, in the form of hernia of this nature.

### IMMEDIATE CAUSES OF THE FATAL ATTACK.

These causes are noticed by writers thirty-four times.

*Table* 12.

| | | |
|---|---|---|
| By a fall | | in 9 cases. |
| " violent emetic | | " 3 " |
| " laxative medicine | | " 1 " |
| " drunkenness | | " 5 " |
| " inordinate use of cabbage and vinegar | | " 1 " |
| " " | cold acidulated water | " 1 " |

| | | |
|---|---|---|
| " excess at a ball . . . . . . | " 1 " | |
| " bullet wound . . . . . . . | " 1 " | |
| " stab . . . . . . . . . | " 3 " | |
| " blow . . . . . . . . | " 1 " | |
| " strain, violent and sudden . . . . | " 1 " | |
| " being run over . . . . . . . . | " 3 " | |
| " violent crying . . . . . . | " 1 " | |
| " labor pains . . . . . . . . | " 2 " | |
| " a long walk . . . . . . . | " 1 " | |
| | 34 cases. | |

In eleven cases the hernia was congenital and almost immediately fatal; making in all forty-five cases in which the causes of fatal attack were given.

### INFERENCES TO BE DRAWN FROM THE ABOVE TABLE.

The table is interesting in many particulars. It may be again classified, whereby we shall bring out four great classes of causes of the fatal attack.

*Table* 13.

| | |
|---|---|
| 1st. Pressure either from internal or external causes, . . | 17 |
| 2d. Indigestible and irritating substances taken into the stomach, | 12 |
| 3d. Wounds, . . . . . . . . . . . . | 4 |
| 4th. Congenital, . . . . . . . . . . | 11 |
| 5th. Great fatigue from a long walk, . . . . . . . | 1 |
| | 45 |

From the second class of causes we have important indications for prophylactic treatment; and of this I shall treat more fully hereafter.

The comparison of the number of fatal results immediately after birth in congenital cases, with the absolute number of congenital cases on our record, proves very conclusively that congenital hernia of the diaphragm is by no means so necessarily immediately fatal in its consequences as some authors would have us believe — nine in twenty-four of those affected with it, or a little more than one third only, having died at birth.

### IV. AGE, SEX, PROFESSIONS, &c., OF THOSE AFFECTED WITH THIS HERNIA.

The average age of seventeen males was $35\frac{16}{17}$, or 36 years nearly, the oldest being 60 and the youngest 19.

Of three females the average age was 25 years, the oldest being 28, the youngest 19 years. In addition to these, we have the indefinite but expressive phrase "old woman" applied to one of the patients.*

There were four under 7 years of age, with sex unknown, and nine still-born infants, sex, likewise, unknown; finally six, whose age and sex are alike unknown.

The relative number of the *sexes* was as follows: out of seventy cases in which the sex is named, there were

| | |
|---|---|
| Of males, | 53 |
| Of females, | 17 |

It may be a question why there should be this great difference between the sexes in their liability to this disease. A glance at table 13 will readily solve the question. We see that of the four classes of causes, only two apply with equal force to males and females, viz., second and fourth; whereas the other two, viz., first and third, are almost exclusively liable to affect the male sex, from the fact that the males are the laborers and warriors of the race.

The *professions* of those suffering from this complaint, are interesting as indicating the amount of real deterioration to health caused by it. In only twenty-five cases is any record made, and the result is as follows:

*Table* 14.

| | |
|---|---|
| Common soldiers, | 7 |
| Officers, (military,) | 3 |
| Masons, | 2 |
| Shoemakers, | 2 |
| Sailors, | 4 |
| Husbandmen, | 1 |
| Slater, | 1 |
| Conductor of Diligence, | 1 |

* Since this was written I have found the case of a female, aged eighty. It is evident, therefore, that a greater number of facts is needed before we can decide the question.

| | |
|---|---|
| Postillion, . . . . . . . . . . | 1 |
| Laborer, . . . . . . . . . | 1 |
| Carpenter, . . . . . . . . . | 1 |
| Student, . . . . . . . . . | 1 |
| | 25 |

| | | |
|---|---|---|
| *Total.* | Laborious professions, . . | 22 |
| | Light do. (student and shoemaker,) | 3 |
| | | 25 |

Ten of these twenty-five died in consequence of their injuries, either instantly, or after a few days. The remainder lived a greater or less time afterward, and performed some of the most laborious duties of life. For example: a sailor went several India voyages; a husbandman died at a good old age; a postillion cracked his whip for ten long years, and at length died of a second fall; a shoemaker kept himself busy at his last for a great while; all enjoyed a comfortable degree of health but were liable to those symptoms to which I have alluded in another part of this paper. (See symptoms antecedent to fatal attack.) Our hospital case was a striking illustration of the fact that a man may have diaphragmatic hernia and yet be able to perform much hard work. He was in fact a most beautifully proportioned youth, a sort of Antinous, as he appeared when his trunk and limbs were laid bare during the examination of his fractured spine. All his muscles were fully and gracefully rounded, and their undue prominence was prevented by a certain quantity of adipose matter indicative of perfect health.

### V. DURATION OF LIFE.

I shall consider this subject under two points of view, viz., the duration of life in congenital and accidental cases. Under the latter term I include all except the congenital.

There were twenty-six *congenital* cases, and of these persons

*Table* 15.

11 died within two hours after birth.
6 within two years.
1 at seven years.
8 not until the adult age.

Duration of life in the *accidental* cases, thirty-one in number.

Death was *instantaneous* in four cases; (arising, one from falls; one from a severe blow; one from a violent and sudden strain; one from labor pains.)

It took place within twenty-four hours in twelve cases; (viz., two from wound; one from fall; two from being run over; one being beaten and drunk; one from drunkenness; one from crying violently; two after an emetic; one from cabbage taken; one from labor pains.)

It occurred within a week in seven cases; (one from a stab; two from a fall; one being run over; one cold water; two from drunkenness.)

It took place within a year in five cases; (two from wound; one from drunkenness; two from falls.)

Life lasted more than a year in three cases; (viz., two from wounds; one from a fall.)

In one of these last cases life was prolonged thirty-eight years.

The shortest time was about two years.

It will be seen by the above statements that

28 died within a year after the accident.
3 lived beyond that period.

This proves, conclusively, that accidental diaphragmatic hernia is much more liable than congenital soon to destroy life. Or to express the same in in numbers: about one-tenth live more than a year in accidental cases; a little more than one-half live beyond that period in congenital cases.

### VI. DURATION OF LIFE IN THE FATAL ATTACKS.

In twenty-seven cases the duration of the attack is mentioned. The average of all of these is two days and fifteen minutes. The shortest time was, of course, instantaneous death; the longest was nine days. The causes that operate the most quickly are those which produce sudden and violent strains, such as blows, falls, &c.: the next in order may be ranked all indigestions, from having taken too much of substances that are prejudicial to the stomach. But it is impossible to make a definite classification. The average duration proves the fearfully rapid course of the fatal attack.

### VII. DIFFERENT SPECIES OF HERNIA.

Sir Astley Cooper* made three species, and that division has been usually

* On Hernia. London. Fol. 1804.

followed by authors, in England and on the continent, since his days. They are as follows:

1st. In the first species we find the parts, of which the hernia consists, are forced through some one of the natural openings of the diaphragm; for example, that of the aorta, vena cava inferior, an intercostal nerve or the œsophagus. May we not also consider the sacs, of which I have heretofore spoken, as the result of enlargement of small apertures naturally existing between the anterior mediastinum and the cavity of the abdomen? It seems to me as reasonable to classify this set of facts under this head, as it is to put those cases there in which it is said that an intercostal nerve passage is dilated.

2d. In this species, are included all hernias resulting from a malconstruction of the diaphragm.

3d. Hernias from accidental wounds or lacerations of the diaphragm.

To these I would add a fourth kind; although, in the opinion of some, the cases are not, strictly speaking, cases of hernia.

4th. Those cases in which one side of the diaphragm is violently forced up into the chest, so that the lung is compressed, and all the signs of the affection, as seen in the other classes, are observed.

In a former part of this paper I have investigated some of the points connected with this subject.

Of the relative frequency of these four classes, I obtain from sixty-eight cases the following:

*Table* 16.

| | |
|---|---|
| Hernia from accidents, | 41 |
| " " malconstruction, | 20 |
| " " dilatation of natural openings, | 5 |
| " " diaphragm being pushed up, | 2 |
| | 68 |

These numbers agree with the opinions advanced by Sir Astley Cooper. Of the first three, he says, the third is of very rare occurrence. He had never seen one.

The second kind he says is more frequent.

The first kind is the most common, but as most of the accidents are, in the opinion of Sir Astley, caused by the small sword, this class will, of course, ebb and flow according to the character of the age.

### VIII. DIAGNOSIS.

The recognition of this disease has usually been made only after death. Some writers have said that it would be impossible to discover it during life. It has, in fact, been recognized, I believe, but a very few times before death. I find only two recorded instances, viz., that by Mr. Laurence, of London, and our own case, recognized by myself and others, at the Massachusetts General Hospital. The reason for this apparent difficulty in the diagnosis is the extremely infrequent occurrence of the affection. No man can hope to see more than one case during his lifetime, and therefore, as his attention is not drawn to the subject, he is unable to recognize the affection when it falls under his notice. Yet, from the investigation I have made upon the subject, I am disposed to believe that the diagnosis of diaphragmatic hernia is as easy, as that of almost any other chronic, and, possibly I might add, acute disease. The rational signs distinctly point to it, in most cases; the physical signs will generally afford the *experimentum crucis*, and definitely settle the question. In our own case at the hospital, these signs alone proved that the intestines or stomach must be in the left side of the thorax, and if we, as good observers should have done, had compared more accurately than we did the rational with the physical signs, we should, I think, have been compelled to infer that there was a congenital diaphragmatic hernia, instead of a simple rupture of that muscle; which latter condition we were led to believe was the case from the circumstances of severe injury in the back, under which the patient entered the hospital.*

I shall treat of the subject of diagnosis under the following heads:

*First.* Congenital cases.

*Second.* Cases in which a sac is formed.

*Third.* Accidental cases.

### *First.* DIAGNOSIS OF CONGENITAL CASES.

This category of facts may be still further divided, viz: 1st. Into those who die at birth or immediately afterward. 2d. Those who live for a few months, or years, in a state of more or less constant ill health. 3d. Those

---

* Since writing the above, I learn that the house surgeon made this inference from facts in the early history of the patient, but which, from the sufferings of the patient, I had been unable to obtain.

who arrive at the adult age, and are able to perform many of the duties of life, even those, at times, involving the hardest kind of labor.

1st. In the first class, nearly one-half never breathe, nor can they be made to breathe. They die at the moment of their birth. It is literally "death in life" with these little creatures; for, although the placental circulation may have gone on in perfect order, and the infant may be well formed, save in the want of a part, or the whole of the diaphragm, his doom is sealed at the moment of his first effort of inspiration. He can not inspire and he dies instanter, or with a few gasps or sighs. He may make some very feeble attempts at crying; and, in very rare cases, may seem to be recovering; but he will relapse and die within a few hours. The other functions of the body may be, though very imperfectly, performed; the circulation may go on, but the heart should be ausculted, for we shall frequently find it beating out of its wonted place. If it be so, our diagnosis and prognosis may be more decided. The nervous system may sympathize, and shudderings or severe convulsions may occur, to close the scene. If with these symptoms, we find lividity of the skin and evidently general distress, referable to trouble in the respiratory function; if auscultation and the physical signs, already detailed, confirm the rational signs, we certainly may be as sure of our diagnosis, and of the proper method of treatment, as we can be of almost any disease, pneumonia, for example.

In addition to these means of recognizing the affection, I would remark, that any external malformation, such as spina bifida, &c., combined with the above-named symptoms, should lead us to suspect malformation of the diaphragm, upon the same principle that when signs of cephalic disease occur in the course of any malignant affection, that is well marked externally, we infer that the same disease has commenced in the brain. Facts sustain my assertion, several cases of monsters having exhibited this malformation.

2d. Congenital cases in which the patients live months, or a few years, but generally in ill-health. Dyspnœa seems to be the most prominent symptom. It is either constant or the patient is very liable to it upon the slightest exertion; the attacks come on suddenly, and as suddenly leave; sometimes position and clothing have much influence. The whole constitution of the individual seems bad, and the symptoms generally point to a chronic difficulty about the respiratory function; the little patients are at times emaciated and feeble. Auscultation will come to our aid as in the previous case.

3d. There are some who arrive at the adult age, notwithstanding there is a very material malconstruction of the diaphragm. It seems almost impossible that such persons should be able to perform any hard work, but such is the case in not a few instances, and not merely are they able to perform all the work of hard laboring men, but they have at times a degree of *embonpoint.* This was particularly the case with our youth at the hospital, with his fair, ruddy countenance, and rounded swelling limbs and chest. But though thus generally healthy, these individuals are liable, on severe or sudden exertion, to violent, and at times, prostrating attacks of dyspnœa, causing, as in our case, total loss of consciousness. These attacks may come on very suddenly and as suddenly disappear.

The *pulse* in these cases is at times very materially altered, quickened and tremulous, owing to the dislocation of the heart.

*Pain in the side* about the region of the diaphragm, is not uncommon. *Colics* and costiveness are not unusual.

*Vomiting* is frequently seen, and this is a very dangerous symptom to be excited either by an emetic or by improper food; for it aggravates the affection.

But after all, no symptoms of a general nature can, in these cases, be equal in value with the physical signs.

### *Second.* DIAGNOSIS OF CASES IN WHICH SACS ARE FORMED.

Some writers* have hinted that there is a distinct difference between the symptoms of these cases, and of those in which there is a rupture, or a dilatation without a sac through the various layers of the diaphragm. That one may imagine a difference, I will not deny, but that the records of science give any support for such an hypothesis I am disposed to doubt. My reasons for this opinion are that the records are too slight wherefrom to draw any inferences.

### *Third.* DIAGNOSIS OF HERNIA ARISING FROM ACCIDENTAL RUPTURE OF THE DIAPHRAGM.

This class of cases may be divided into those in which 1st. The patient dies immediately or in a very short time after the accident; and 2d. Those in which the individual recovers from the immediate effect of the injury, and rupture is discovered at death, or may be suspected during life, by the symptoms incident thereto.

* Arch. Gen'les de Med., 2d ser. tom. 12, p. 387.

1st. Of those who die in consequence of the accident, some few persons* are found in whom no marked and very severe symptoms come on until after the lapse of some hours after the accident. In all the cases alluded to I have found the men† were drunk, or nearly so, and their faculties were probably so benumbed that they did not experience the ill-effects they would have felt had they been clothed in their right minds and sensibilities. But generally a most violent train of symptoms commences immediately, varying of course somewhat according to the amount and peculiar character of the lesion. Many die immediately after having been so wounded by a bullet, or having fallen from such a height, as to entirely and very promptly destroy life. About an equal number die within a few days; nine days being the longest, and thirty-three hours the shortest period in our cases. In these persons violent breathlessness, orthopnœa, or simple dyspnœa is observed most frequently. With this, pains in the thorax, or either of the hypochondria, are likewise common. Cough is occasionally present but more rarely, causing increased pain in the chest and abdomen. Very rarely is there any expectoration; but at times hœmoptysis or hœmatemesis occurs, that is if either the lungs or stomach are wounded. The pulse is very much quickened and must be altered as in any severe affection of this nature. Vomiting, or ineffectual efforts to do so are frequent, and in the midst of these symptoms, coupled with the prostration that commonly follows a severe internal injury, the patient dies.

2d. At times he recovers from the immediate effects of the injury, owing, probably, to its being of a less severe character than in the cases already considered. In this case the individual may have no untoward symptom, until symptoms of some internal strangulation occur, and he dies, and at the autopsy we shall find the remnants of the former injury in the shape of an abnormal aperture in the diaphragm, and a hernia into the chest of the abdominal organs. This freedom from symptoms, until the fatal attack, is, however, rare, and it is fortunate for humanity that it is so, for when they occur, and do not prove fatal, they may be of infinite service to us in our subsequent treatment of the patient, by enabling us to prescribe certain dietetic and other rules whereby the individual may possibly arrive at a green old age, instead of being cut off in the vigor of manhood, as was the case recently in a very remarkable example.‡ These symptoms are, a liability to dyspnœa

* Mr. Wheelwright's case, Med. Chirurg. Transac., vol. v., page 574. 1815.

† Archives Gen'les de Med. Sept. 1834.

‡ Alexander Barrow, U. S. Senator from Louisiana, we learn from the public papers, on exertion, pains in the hypochondria, or chest; colics, costiveness and vomiting. Here, too, should we use with great care, the physical signs. In one case on record, the heart beat to the right; in ours, likewise, there was the same fact. If, then, we find the heart beating in a different place from usual, and other signs already mentioned indicating diaphragmatic hernia, we must charge our patient to be most cautious, in every action, for the sword of death hangs suspended by a hair over him. The symptoms above stated, may be liable to recur for many years. He may have several attacks of them and may recover, or he may be cut down by the first strangulation of the contents of the hernia, which of course is the cause of the severer attacks, while those symptoms that are habitual, the dyspnœa, &c., are owing to the compression of the lungs, from distention of the stomach and intestines, &c.

Finally, however, death comes on, perhaps after a debauch, an emetic, or another accident, or it may be without any evident cause. The symptoms are the same as those already described as precursors of death, and joined to them we may have hiccough, syncope, cold clammy sweats, and lividity.

### DIFFERENTIAL DIAGNOSIS.

Might not asthma, pneumo-thorax, phthisis, cardiac disease, or a case of that rare disposition of the organs in which the heart is found at the right side, &c., could not common colic, biliary calculi, abdominal hernia, or internal strangulation, simulate diaphragmatic hernia? If we examine carefully the origin, the course and actual state of the symptoms, and do not neglect the physical signs, I can scarcely conceive of a mistake being committed.

*Asthma*, it is true, causes dyspnœa, but the symptoms are excited by a very different cause. The symptoms we recognize, likewise, at once, as the result of a constriction of the bronchi marked by a wheezing, and by physical signs to correspond. Cough, too, is always present in asthma—rather rare in this hernia. Auscultation would allow us to hear wheezing every where in one case, whereas absence of respiration or borborygmi would be discovered in the other.

*Pneumo-thorax* is rarely latent and chronic; it comes on suddenly during phthisis or some similar disease. It has but rarely any abdominal symptoms;

---

died of this internal strangulation, in consequence of an old sword wound of the diaphragm. As we shall see when speaking of the treatment, the journey he was engaged in, the necessary disturbance of the whole system consequent thereupon, might have been foretold to him, had the disease been previously recognized.

the symptoms occur but once, and these are usually fatal. The physical signs may admit of doubt, but usually the borborygmi will settle the matter.

A change in the usual disposition of the organs will not commonly be accompanied by any symptoms. The heart may beat at the right side, but the respiration will be pure throughout the chest.

Simple phthisis or cardiac disease I cannot believe will ever cause any serious difficulty in the diagnosis.

The passage of gall stones may cause severe pains about the diaphragm, but rarely thoracic symptoms. Moreover the physical signs are different in this case and in hernia.

In regard to the supposed similarity of abdominal hernia or internal strangulation, the fact, first, that in the former there would be usually some external tumor; and second, that in both all thoracic symptoms would most probably be absent; and finally, that we have the physical signs very different in the two classes of cases; these facts, it seems to me, would be sufficient for a thorough discrimination of the two affections.

### IX. PROGNOSIS.

We have seen that congenital cases of diaphragmatic hernia are not necessarily, immediately, fatal, the number immediately resulting in death to the whole number of congenital cases, being about one to two four-elevenths. A similar remark may be made in regard to hernia resulting from accidents; for we have found that a little less than one-tenth of all the cases are not fatal, but the patients are alive more than a year after the accident.

But though death may not be the result, we shall, most probably, have a series of symptoms clearly indicative of the disease. Upon this point, however, I must refer to the detailed account of the symptoms and to the chapter on Diagnosis. From these we may infer somewhat as to the probable course of the disease. If we can persuade a man to take reasonable care of the digestive functions, to avoid costiveness; to cease from all very violent exercise or employment; in fact, to do all things in such a way as most to promote quiet health, then our prognosis may be favorable. If, unfortunately, he should be taken ill, we should avoid all violent means, especially those operating severely on the alimentary canal. If these precautions be observed, we may enable a person to enjoy a comfortable degree of health; and in case of actual strangulation, afford him a tolerably fair chance of recovery. If, on the contrary, the individual is of irregular habits, indulges his palate, and is generally of a dissipated life, our prognosis must be unfavorable.

With regard to the result of the actual attack, the severity of the local symptoms and their effects upon the whole system, must be our guide. For example, as long as the vomiting and costiveness, and pulmonary symptoms are slight, and the pulse and powers of the body are not prostrated, we may give some hope. But if the dyspnœa is intense, the vomiting stercoraceous, the pains about the chest or diaphragm agonizing; if the pulse become frequent and feeble; if the skin become covered with a clammy sweat; if the strength of the patient fail, our prognosis must be very unfavorable.

Finally, it must be remembered that all I have stated above is closely allied to, and may, in fact, be considered as absolutely dependent upon an accurate diagnosis. This has never been made but twice, and in these cases it was too late to do service. We must do better than our predecessors, better, likewise, than we ourselves have done, and recognize the affection before the commencement of its fatal attack. This, as we have seen, can undoubtedly be done, if we will but be accurate in our examination of the rational and physical signs, and connect them with previous facts in the history of the patient in the way as indicated in various portions of this paper.

### X. TREATMENT.

In considering this part of our subject, I shall rely little upon the analysis of the results of treatment in the individual cases recorded; but rather upon the suggestions that have arisen in consequence of the study, that I have made, of the whole course of the affection. The reasons for so doing are these: 1st. The small number of cases in which any records are made of any treatment. In only thirteen, in fact, is there any record. 2d. Owing to the ignorance of most of the observers in regard to the true nature of the affection, their modes of treatment have been entirely empirical, and generally very absurd, and not a few times absolutely hurtful to the patient. Occasionally, a course of action, having a good influence, has been commenced, but owing to this same ignorance of the actual morbid condition, all the good has been destroyed, by subsequent exhibitions of remedies of a most pernicious tendency. The consideration of this question of treatment still further naturally divides itself into the *prophylactic*, and that to be followed during an attack. Of the two branches of the subject, I consider the former the more important.

The *prophylactic* mode of treatment, which I shall lay down, will, of course, depend wholly upon what we have learned of the condition of the parts in this affection, and of the tendencies to their strangulation under various circumstances. I have already alluded to this when speaking of the prognosis. Let me suppose either that a child present, from the moment of birth, signs of this hernia, or that an adult is liable to fits of dyspnœa, or costiveness, &c., (see Diagnosis,) after a fall, or a wound in the trunk from a sword or bullet, &c. Let me suppose that the physical signs, inspection, have enabled us to decide which side of the diaphragm the rupture exists, what course are we to advise our patient to pursue, in order to avoid the risk of a fatal attack of strangulation?

If we turn to the list of causes of the fatal attack, we shall see that there are two conditions of the system which are particularly liable to produce serious effects, viz: 1st. Any condition that would be likely to produce violent, or sudden, or long-continued strain on the abdominal muscles; and 2d. Any ingesta taken into the stomach which excites it into undue action. We should therefore advise the patient to give up the practice of any employment likely to produce the first result; for example, that of a seaman, a soldier, or coach dirver, or even of common laborer. He should select some more quiet, though not sedentary employment, as the least liable to produce the results that are anticipated. This treatment I recommend, also, because, from Table 14, I learn that the laborious professions seem more liable to this disease than should be their proportion supposing all equally liable to it. But, at the same time, that he may choose a more quiet business, he must be very careful not to select one liable to produce irregularity of the digestive operations; for instance, that of a tailor would not be very good for a man; and the same remark may be made in regard to the business of a seamstress in the case of a female. In both of these professions dyspepsia, and its train of evils, costiveness and vomiting, &c., are apt to occur, and if they do, the patient will narrowly escape with his life. The business of baker, bricklayer, grocer, servant, merchant, or either of the so-called, liberal professions, would be the better.

2d. We should advise the patient to be very careful that his digestive functions go on well; we should assure him that by a debauch he runs the risk of his life; and that by undue eating of unusual food of any kind, a danger is incurred which no wise man would incur for the mere gratification of the palate. He should use that food which is easiest of digestion, and which has neither a constipating nor a purgative effect; but which will enable him to have regular and sufficient and easy alvine discharges daily. These remarks are suggested by Table 12. It may be further remarked while upon this subject, that on no account should emetics or cathartics, of a violent nature, be prescribed for one having this hernia; for antimonial emetics, that is, *any violent emetics* are very liable to produce death in these cases.

The treatment of a patient suffering from symptoms of actual strangulation, is less satisfactory than one could wish. There are records in thirteen cases only, and even if there had been notes of a greater number, they would hardly have taught us much, for every case on record, in the annals of medicine, has proved fatal, which fact presents a most conclusive argument that, up to the present hour, at least, no course of medication has been of any avail, or if it has been, no medical record at present existing, can prove it to have been so. But are we to infer from this state of things, that we shall never be able to cope with this affection? By no means. When, by our more accurate diagnosis we shall be able to recognize, *early* in the period of strangulation, we may, by cautious treatment, be able to give the patient a fairer opportunity of recovering, than nature, unassisted by art, would afford him; and certainly a far better chance than the various empirical methods pursued by all previous practitioners.

I shall speak of the various remedies that have been used, and give my own views upon the advantages, or disadvantages likely to result from each.

*Venesection* was used five times, and never with any effect. On the contrary, the distressing symptoms went steadily on till death. The amount of blood taken seems never to have been very great, only once is it stated that ℥xvi. were taken. Are we to infer from this result that venesection is never to be performed? I think not. The cases are too few in number to authorize such a deduction, especially as it does not appear that in any of the cases deliquium was produced. Now to do much good, *i. e.* to produce any material relaxation of the strangulation, it seems to me a decided effect should be produced. At the same time, however, there are serious objections to its use, viz., we deprive a man of a great portion of his life's strength by venesection, and we do so without much hope of good. I think, however, we should be justified in bleeding ad deliquium, a stout patient, who is suffering much pain, and who has a strong pulse; but we should be chary of such a method in the cases of more feeble persons.

*Leeches* were used once without relief, and I do not believe they ever would do much good.

*Cathartics and enemata* were given four times. This is, likewise, a small number of cases whence to make any deduction. Yet I think a fair examination of these few, and some, *a priori*, reasoning on the subject, will lead us wholly to reject these two methods as something worse than useless.

The patients were never relieved by either of the methods. On the contrary, all were great sufferers from violent spasms, or colics, or vomiting; and in one, at least, the vomiting was excited by the medicine, and in another, the pains in the abdomen were more severe after their use. And we may fairly, *a priori*, ask the question: Is not this exactly the result we should anticipate? Does an able surgeon give cathartics in a case of strangulated inguinal or crural hernia? To force, by the peristaltic action, more of the contents of the intestine into the strangulated part would be mischievous? Why, then, should we use cathartics and enemata in hernia of the diaphragm? There is no reason, but our ignorance and our disposition always to play the hero in any case of obscure abdominal trouble resembling colics. If a man does so, he is liable to be the cause of the destruction, perhaps, of human life. I am, therefore, fully of the opinion that all cathartics, even of the gentlest kind, should be avoided. The only hope for the patient rests on the entire quiet which the intestinal canal preserves.

*Emetics.* Some may doubt about the truth of the assertion that the physician may be the cause of death by the administration of cathartics. I feel perfectly certain that such would be the case in the use of emetics. If we look at the table of causes, (Table 12,) we find that a violent emetic was the cause of the fatal attack in three cases. In the single case in which it was mentioned as having been used as a remedy, the dyspnœa became much greater after it, and death took place an hour after its exhibition. Taking these facts into consideration, that vomiting is a very troublesome symptom of the disease, I think we are fairly justified in concluding that emetics, not only do no good, but that they tend to aggravate the affection, and, therefore, should never be administered.

*Opiates* were used four times. In one case, where they were freely administered, the patient lived until the sixth day. Though one case affords but small data, whereupon to build an opinion, still as this was a severe case, and continued longer than the average number of days before death, it seems to me to be of some importance. In the other cases in which opiates were administered, they were given in very small quantities and without any definite aim; so that no marked effect was produced, save in the relief of pain.

But does it not seem reasonable that they should be of service in preventing any undue peristaltic action of the bowels, while we are using other remedies for the relief of the inflammatory condition of the ring and parts adjacent. Thus the analysis of facts and reasoning, *a priori*, lead us to conclusions exactly the reverse of those to which the subject of cathartics has brought us.

Other remedies of a general and very indefinite nature, are mentioned by authors, such as "soothing antiphlogistics;" and "stimulants afterward," "remedies for spasm of the stomach;" "creosote," "vesication and sinapism" to side. But of these I shall say nothing.

*Bathing* was used once, for a quarter of an hour, with relief. But should it not be used more freely? Nothing that I know of has a more powerful effect on the system than long-continued bathing. It evidently relaxes all parts with which it comes immediately in contact, and I think that the cold bathing, as used by the hydropathists, would tend, at least in a degree, so to lower the pulse, and diminish any local inflammatory tension even of the diaphragm, that it is worth a trial.

*Ether.* Were I satisfied that stricture existed in the diaphragm, I should be disposed to use this new method freely. It would do no harm and would temporarily relieve the pain and give relaxation to all the parts, and thus give a chance, at least, of active relief to the strangulation.

*Operation.* Finally, as a last resource, might not an operation for cutting the strangulated ring be attempted? It never has been done, though Laennec has suggested it. Yet I see no good reason why it may not be possible to recognize, by the physical signs, the side at which the disease exists, and the probable amount of the affection. Having learned these two points, and having tried other means of relief without success, ought we not to undertake the more serious operation of the scalpel?

Where should the incision be made? Sacs commence about the lower and front part of the mediastinum; therefore, near this is a proper place to commence the incision. Passing along the edge of the ribs for the space of three or four inches, we should divide the muscles and come upon the peritonæum covering the lower part of the diaphragm and reflected thence upon the inside of the abdominal muscles. It would be possible, (I think,) to push aside this membrane and not enter the cavity of the peritoneal sac until we might be able to make out the exact place of stricture, and there a very slight incision only would be necessary. But in this operation there is, at present, so much of difficulty owing to the proximity of important organs, and to the distention and alteration of position of the abdominal organs, that it will probably have few to perform it, especially as the disease is so rare, that no person would be likely to have more than one or two opportunities for operating during his whole lifetime.

List of works from which the above cases were taken, or which were consulted during the preparation of the monograph:

1610. Opera Chirurgica. Ambrose Paré. Frankfort.
1646. Gulielmi Fabricii Hildani, opera. Frankfort.
1698. Lazari Riverii Op. Medica Univers. Obs. 67.
1702. Philosophical Transactions of the Royal Society of London; abridgment. Paper by Sir Charles Holt.
1729. Mémoires de l'Académie des Sciences. Article by Chauvet & Senac; also 1772, by Vicq d'Azyr. Paris.
1755. Haller's Dissertationes Chirurgicæ, vol. iii. Dissertatio de Hernia Ventriculi; by Kirschbaum. Lausanne.
Morgagni Works, vol. iii., letter 54. Seats and Causes of Disease.
1767. Historia Anatomico-Medica; Josephus Lieutaud. Paris, vol. i.
1771. Medical Observations and Inquiries. London.
1784. Works of John Fothergill. Edited by J. R. Lettsom. London.
1803. Cours d'Anatomie Médicale. Antoine Portal. Paris.
1804. On Hernia. Sir Astley Cooper. London.
1807. Edinburgh Medical and Surgical Dictionary; by Morris & Kendrick.
1815. Medico-Chirurgical Transactions. London.
1818. Dictionnaire des Sciences Médicales. Paris. Percy on Rupture of the Diaphragm.
1823, '26. Edinburgh Medical and Surgical Journal, vol. xix., p. 293 and 382.
1823. Supplement au Traité Pratique des Hernias, par Scarpa. Paris.
1825. Revue Médicale et Journal de Clinique, vol. xvii. Paris.
1827. Journal des Progrés Médicales. Do., 1828.
1828. Medico-Chirurgical Review, vol. ix., p. 280.
1831. London Lancet, April; Nov., 1834; Sept., 1835; April, 1840; Aug., 1843.
1832, '3, '41. London Med. Gaz., vol. x., p. 42; vol. xii., p. 673; vol. xxviii., p. 390.
1834. Dissertatio Inauguralis Anatom. Chirurg. de Hernia Diaphragmatis. Hubertus; Griffiœn; Stierling. Heidelberg.
1835. Dictionnaire des Sciences Médicales, in 30 vols. Paris. Art. Diaphragme. Cloquet & Berard.
1838. Treatise on Hernia; by William Lawrence. London.
1839. Archives Generales de Medecine, 3d ser., 19 vols.; vol. vii.; vol. xviii.
1842. These pour le Doctorat en Medecine, par Aristide Auzelly. Paris.
1846. Bulletin de la Societé Anatomique. Case by Mons. Gabier.
Philadelphia Medical Examiner, vol. iii., p. 384.
British and Foreign Medical Review, vol. iv., p. 260.
Encyclographie des Sciences Méd. Ser. 6, Tom. 12, p. 267.

In addition to the above works, from which I have obtained the cases, I have examined all the medical and scientific journals and transactions which I could find up to the date of the paper, together with all treatises upon the subject of hernia, to which I could obtain access.

